# Diabetes-specific genetic effects on obesity traits in American Indian populations: the Strong Heart Family Study

**DOI:** 10.1186/1471-2350-9-90

**Published:** 2008-10-14

**Authors:** Nora Franceschini, Laura Almasy, Jean W MacCluer, Harald HH Göring, Shelley A Cole, Vincent P Diego, Sandra Laston, Barbara V Howard, Elisa T Lee, Lyle G Best, Richard R Fabsitz, Kari E North

**Affiliations:** 1 Department of Epidemiology, University of North Carolina, Chapel Hill, NC, USA; 2 Department of Genetics, Southwest Foundation for Biomedical Research, San Antonio, TX, USA; 3 MedStar Research Institute, Washington, DC, USA; 4 Center for American Indian Health Research, College of Public Health, University of Oklahoma Health Sciences Center, Oklahoma City, OK, USA; 5 Missouri Breaks Industries Research, Inc., Timber Lake, SD, USA; 6 Epidemiology and Biometry Program, National Heart, Lung and Blood Institute, Bethesda, MD, USA; 7 Center for Genome Sciences, University of North Carolina, Chapel Hill, NC, USA

## Abstract

**Background:**

Body fat mass distribution and deposition are determined by multiple environmental and genetic factors. Obesity is associated with insulin resistance, hyperinsulinemia, and type 2 diabetes. We previously identified evidence for genotype-by-diabetes interaction on obesity traits in Strong Heart Family Study (SHFS) participants. To localize these genetic effects, we conducted genome-wide linkage scans of obesity traits in individuals with and without type 2 diabetes, and in the combined sample while modeling interaction with diabetes using maximum likelihood methods (SOLAR 2.1.4).

**Methods:**

SHFS recruited American Indians from Arizona, North and South Dakota, and Oklahoma. Anthropometric measures and diabetes status were obtained during a clinic visit. Marker allele frequencies were derived using maximum likelihood methods estimated from all individuals and multipoint identity by descent sharing was estimated using Loki. We used variance component linkage analysis to localize quantitative trait loci (QTLs) influencing obesity traits. We tested for evidence of additive and QTL-specific genotype-by-diabetes interactions using the regions identified in the diabetes-stratified analyses.

**Results:**

Among 245 diabetic and 704 non-diabetic American Indian individuals, we detected significant additive gene-by-diabetes interaction for weight and BMI (*P *< 0.02). In analysis accounting for QTL-specific interaction (*P *< 0.001), we detected a QTL for weight on chromosome 1 at 242 cM (LOD = 3.7). This chromosome region harbors the adiponectin receptor 1 gene, which has been previously associated with obesity.

**Conclusion:**

These results suggest distinct genetic effects on body mass in individuals with diabetes compared to those without diabetes, and a possible role for one or more genes on chromosome 1 in the pathogenesis of obesity.

## Background

Body fat mass distribution and deposition are determined by multiple environmental and genetic factors. Obesity is associated with insulin resistance, hyperinsulinemia, and incident type 2 diabetes [1,2]. Moreover, insulin has anabolic effects on fat metabolism leading to fat deposition and obesity [3]. Obesity and diabetes are highly prevalent in American Indian populations. Among individuals between 45 and 74 years who were recruited into the Strong Heart Study, approximately 35% were obese [4] and type 2 diabetes prevalence varied by recruiting center from 33% in South and North Dakota men to 72% in Arizona women [5]. The estimated age-adjusted rates of diabetes were higher in recruiting centers with a higher prevalence of obesity [5].

Diabetes and obesity are associated with increased morbidity and mortality in the general population [6] and among American Indian subjects, and they account for increased incident hypertension [7], and fatal and nonfatal cardiovascular disease (CVD) events [8]. The identification of genes for obesity in the environmental and genetic milieu of type 2 diabetes may bring us one step closer to the identification of the functional genes that influence susceptibility to obesity, a major public health problem among American Indian populations.

The genetic component of obesity-related traits among American Indians has been previously described [9-11]. In our previous work [11], we identified evidence for distinct genetic effects on obesity related traits in diabetic compared to non-diabetic Strong Heart Family Study (SHFS) participants. A significant additive genotype-by-diabetes interaction was observed for body mass index (BMI), waist-to-hip ratio (WHR) and percent body fat. The purpose of the present study is to localize these genetic effects on obesity-related traits while accounting for interaction with diabetes status.

## Methods

The SHFS recruitment and genotyping methods have been described elsewhere [12,13]. Briefly, American Indian subjects from 13 tribes were recruited from centers located in Arizona, Oklahoma and North and South Dakota. Clinical information and laboratory measures were obtained during a study exam. Anthropometric measures of body weight (kg) and height (m) were used to estimate BMI (kg/m^2^). WHR (in cm) was also recorded. Percentage body fat was measured using Quantum II bioelectric impedance (RJL Systems, Clinton Township, MI) [12]. The percentage of body fat was calculated using a formula based on total body water [13]. Type 2 diabetes was defined as a fasting blood glucose of 126 mg/dl or higher, history of diabetes or use of diabetic medications [14]. The SHFS protocols were approved by the Indian Health Service Institutional Review Board (IRB), the IRBs from participating institutions and by the 13 tribes participating in these studies. The study was conducted in accordance with the principles of the Declaration of Helsinki. Written consent was obtained from all individuals of this study.

Participants, who are members of extended families, were genotyped for approximately 400 microsatellite markers spaced at average 10 cM intervals across the autosomal chromosomes. Pedigree relationships were verified using the Pedigree Relationship Statistical Tests program [15], which employs likelihood-based inference statistics for genome-wide identity-by-descent (IBD) allele sharing. Mendelian inconsistencies and spurious double recombinants were detected using the SimWalk2 program [16]. The overall blanking rate for both types of errors was less than 1% of the total number of genotypes for Arizona, Dakota and Oklahoma. Marker allele frequencies were estimated using maximum likelihood methods estimated from all individuals [17] and multipoint IBD sharing was estimated using Loki software [18].

We conducted a genome scan of obesity traits while modeling interaction with diabetes status using maximum likelihood methods implemented in SOLAR 2.1.4. We studied 976 individuals from extended families enrolled between 1998 and 1999 (SHS phase 3). We examined the distribution of the obesity-related quantitative traits of body weight, BMI, WHR and percentage of body fat, and excluded observations greater than 2.5 standard deviations above or below the mean (< 5 observations excluded). The traits were adjusted for the effects of age, age^2^, sex, age-by-sex interaction and study center using linear regression models and an alpha = 0.05. The WHR trait was scaled for analysis by multiplying by 10.

We examined the evidence for additive genotype-by-diabetes interaction on obesity traits using maximum likelihood variance decomposition methods extended to allow for the genetic covariance between diabetic and non-diabetic subjects [19,20]. We tested the hypothesis of interaction using the likelihood ratio test comparing a model including genotype-by-diabetes interaction to restricted models in which such interaction is excluded. We estimated the genetic correlation between diabetic and non-diabetic individuals (ρ_G_) and the genetic standard deviations for diabetic and non-diabetic subjects. To test for differential additive genetic effects among diabetic and non-diabetic subjects, we compared a model in which ρ_G _(diabetic, non-diabetic) =1 to a model without constraints. To test for differences in the magnitude of the genetic effects among diabetic and non-diabetic subjects, we compared a model in which the genetic standard deviations of the two groups are constrained to be equal to a model without these constraints. We considered evidence for genotype-by-environment interaction if the genetic correlation between diabetics and non-diabetics was different than 1 and/or the genetic variance for obesity traits in diabetics was significantly different than in non-diabetics.

We also tested for QTL-specific diabetes-by-obesity interaction. For these analyses, we compared a model in which the QTL standard deviations are constrained to be equal, to a model without constraints, using the likelihood ratio statistics. When comparing models with variances constrained to be equal, interpretation of significant differences were based on the assumption of an asymptotic chi-square distribution for the likelihood test statistic. For models that restricted the genetic correlation to one (the upper boundary of the parameter space, i.e., ρ_G _= 1.0), the test statistic is asymptotically distributed as a 50:50 mixture of a chi-square and a point mass at 0 [19]. The gene-by-environment analysis has an additional QTL variance component compared with the standard linkage model, therefore we corrected for the increase in degrees of freedom and all reported LOD scores are corrected LOD score [21].

For the obesity traits showing significant additive genotype-by-diabetes interaction at alpha = 0.05 (ρ_G _≠ 1), we performed genome scans accounting for the diabetes interaction. We adjusted the obtained LOD scores for the difference in the number of parameters between the polygenic and linkage versions of the interaction model, taking into account that each of the QTL standard deviations has a boundary at the null hypothesis.

We used the University of California Santa Cruz  and the miRBase database  websites to search for candidate genes and microRNAs.

## Results

Over 10,000 relative-pairs were available for analysis (Table 1). Approximately 57% of participants were obese (BMI > 30 kg/m^2^) and 26% had diabetes (Table 1). Individuals with diabetes had higher mean weight, BMI, WHR and percentage of body fat than those without diabetes. Approximately 62% of diabetic subjects were using oral hypoglycemic drugs or insulin therapy.

**Table 1:** Descriptive characteristics of American Indian subjects by diabetes status

	Overall participants	Diabetic individuals	Non-diabetic Individuals
Number	976	255	721
Mean age (SD)	42 (16)	53 (14)	38 (15)
Sex female/male	563/400	156/94	407/306
Mean Weight (kg) (SD)	88.7 (20.9)	91.4 (20.4)	87.7 (21.0)
Mean BMI (kg/m^2^) (SD)	31.8 (7.0)	33.7 (6.6)	31.1 (7.0)
Mean % body fat (SD)	36.5 (9.8)	38.4 (8.6)	35.9 (10.1)
Mean WHR (SD)	0.93 (0.09)	0.97 (0.06)	0.92 (0.09)
Number of relative-pairs	10,271	598	6,303

BMI, body mass index; WHR, waist-to-hip ratio; SD, standard deviation

We detected significant additive genotype-by-diabetes interaction for weight (ρ_G _≠ 1, *p *= 0.01), BMI (*p *= 0.0002) and WHR (*p *= 0.02), suggesting that at least some of the genes influencing these obesity traits differed in diabetic compared to non-diabetic subjects (Table 2).

**Table 2:** Additive genotype-by-diabetes interaction for obesity related quantitative traits in American Indian participants of the Strong Heart Family Study

				Genetic standard deviation		
				
Obesity traits	Variance due to covariates*	Residual kurtosis after covariates	Genetic correlation (ρ_G_) ± SE among diabetics and nondiabetics (*P*-value)	Diabetic individuals	Non-diabetic individuals	*P*-value**
Weight	0.11	0.55	0.56 ± 0.16 (0.01)	15.56	18.83	0.26
BMI	0.09	0.75	0.45 ± 0.17 (0.0002)	6.26	5.56	0.50
% FAT	0.47	0.96	0.67 ± 0.18 (0.08)	5.58	7.46	0.16
WHR†	0.22	0.57	0.72 ± 0.15 (0.02)	5.77	5.51	0.77

BMI, body mass index; WHR, waist-to-hip ratio; FAT, body fat; SE, standard error. * age, sex, center; ** *P*-values for the likelihood ratio comparing a model in which the genetic variances for obesity traits in diabetics and non-diabetics are equal to a model without constrains. †The trait was scaled by multiplying by 100.

**Figure 1:**
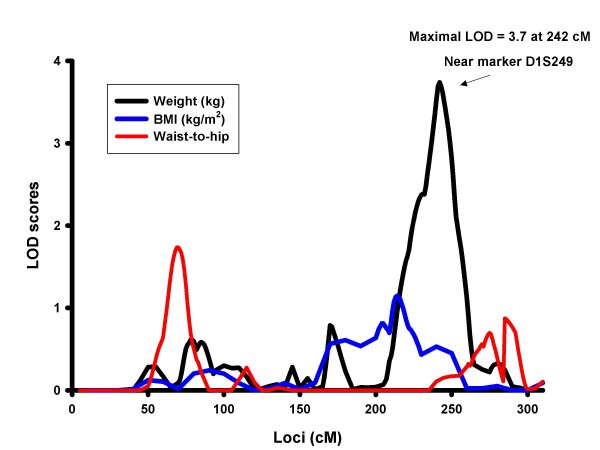
**Multipoint LOD Scores on Chromosome 1 for Obesity Traits Accounting for Diabetes Interaction**. Adjusted for age, age^2^, sex, age-by-sex interaction within centers.

**Figure 2:**
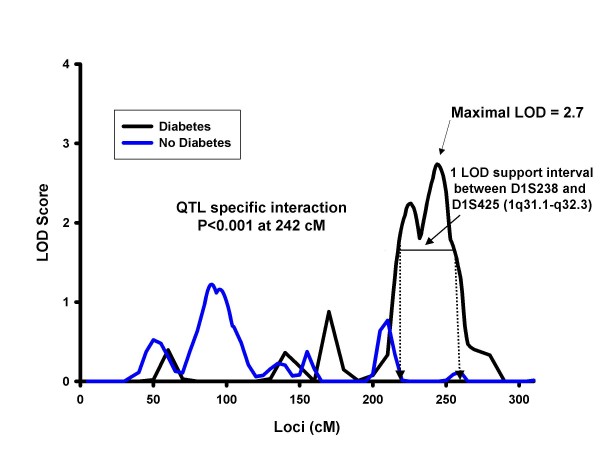
**Multipoint LOD Scores on Chromosome 1 for Weight by Diabetes Status**. Adjusted for age, age^2^, sex, age-by-sex interaction within centers.

Analysis accounting for the genotype-by-diabetes interaction identified a QTL for weight on chromosome 1 at 242 cM (marker D1S249, LOD = 3.7) (Figure 1). For comparison, the diabetes-specific signal at 1q32 for other obesity-related traits is also shown in Figure 1. Stratified analyses by diabetes showed that the chromosome 1 signal was more prominent in diabetic subjects (LOD = 2.7) (Figure 2). The 1-LOD support interval was between D1S238 and D1S425 covering the regions 1q31.1-q32.2, and approximately 23.8 MB. Additional QTLs identified in our linkage analysis of obesity traits accounting for diabetes status are reported in the Additional file 1.

We then evaluated the evidence for genotype-by-diabetes interaction at the QTL level, using the combined samples of diabetic and non-diabetic individuals and the maximum LOD score QTL identified on the diabetes-stratified analysis. The QTL- specific genotype-by-diabetes interaction was significant for weight on chromosome 1 at 242 cM (*p *for interaction < 0.001).

## Discussion

This study identified a diabetes-specific QTL for body weight on chromosome 1 among American Indians, which was less prominent for BMI and for the fat distribution trait of WHR. Diabetes and obesity are strongly associated in American Indian and other populations, and environmental factors such as diet and lifestyle play an important role in both conditions. Our findings suggest a role for diabetes-specific genes influencing body fat deposition in American Indians. Further studies are required to explore the generalizability of these findings to populations of different ethnic/racial and cultural backgrounds, in which the prevalence of obesity and diabetes vary from American Indians. Nevertheless, our findings suggest that the genetic susceptibility to obesity and type 2 diabetes may share common genetic pathways, by complex interactions of genes influencing obesity in the milieu of diabetes.

Several prior studies have showed linkage to the chromosome 1 region for diabetes-related traits and obesity [22], although genetic interactions have not been explored at this QTL. The 1q32 region was identified in linkage analysis of type 2 diabetes in African American participants of the Hypertension Genetic Epidemiology Network (HyperGEN) (LOD = 1.9) [23]. Among non-diabetic individuals, linkage to this QTL was described in studies of fasting glucose among Pima Indians (LOD = 1.5) [24] and among participants of the Framingham Offspring Study (LOD = 1.8) [25], and in studies of fasting insulin among HyperGEN males (LOD = 2.1) [26]. In addition, a West African study of type 2 diabetic sib-pairs described a LOD score of 1.6 for BMI and 1.5 for fat mass (estimated by bioelectric impedance), between the 1q25.1-q31.1 regions, which are within the 1-LOD score support region of our linkage peak [22]. Participants in this West African study were about 5 BMI units heavier than the population from which they were enrolled [22].

Within the 1-LOD support region lays the *ADIPOR1 *gene. The adiponectin 1 receptor mediates adiponectin effects on fatty acid oxidation and glucose uptake through activation of AMP-activated protein kinase and peroxisome proliferator-activated receptor-α [27]. *ADIPOR1 *deficient (-/-) mice show increased adiposity and decreased glucose tolerance, associated with decreased locomotor activity and energy expenditure [27]. *ADIPOR1 *variants have been associated with type 2 diabetes, pre-diabetes states and measures of obesity although not in all studies [28-33]. For example, among Caucasian obese individuals (mean BMI 32 kg/m^2^) with impaired glucose tolerance, *ADIPOR1 *SNPs were associated with measures of body size including weight, waist, hip circumference and BMI [30]. In addition, a gene-gene interaction of *ADIPOR1 *and *ADIPONECTIN *was observed in increased overall and abdominal adiposity and higher substrate oxidation [34]. Therefore, *ADIPOR1 *is a potential candidate gene for pleiotropic effects on obesity and diabetes susceptibility in humans.

It is difficult to gauge the LOD score evidence reported herein in the face of the number of comparisons made in conducting gene-by-diabetes genome scans. In total, we conducted three genome scans (a minimum model, a maximal model and an interaction model). If we were to use a too conservative Bonferoni correction, we would need to observe a P = 0.05/3 * 380 markers: P = 0.00004 or a corresponding LOD score of 3.7. However, given the correlation and non-independence of the three genome scans of each obesity trait, a systematic correction would be extremely difficult.

Multiple interacting genes may underlie this QTL on chromosome 1, and therefore, densely genotyping this region is the next logical step to be taken to identify these genes influencing obesity in the context of diabetes. Our findings are limited to American Indian populations and should be validated in replication samples of other populations.

## Conclusion

In summary, we identified a diabetes-specific QTL on chromosome 1 at 242 cM for weight in American Indians, in the region where the *ADIPOR1 *gene is located. This gene has been associated with multiple measures of body size and diabetes-related traits in humans and experimental models and, therefore, it is likely a good candidate for susceptibility to fat deposition in diabetic individuals. Importantly, our findings support that this region of the genome likely harbors susceptibility variants for obesity that are importantly different in type 2 diabetes individuals and those with normal glucose metabolic profile. Thus, future studies should more comprehensively evaluate the *ADIPOR1 *gene, especially with respect to the complex relationship between body weight and type 2 diabetes.

## Competing interests

The authors declare that they have no competing interests.

## Authors' contributions

NF, JWM and KN participated in the design of the study and drafted the manuscript. NF and HHHG performed the statistical analysis. SAC and SL carried out the molecular genetic studies. VPD, BVH, ETL, LGB and RRF contributed with acquisition of data and revision of the manuscript. All authors read and approved the final manuscript.

## Pre-publication history

The pre-publication history for this paper can be accessed here:



## Supplementary Material

Additional file 1 **Strong Heart Family Study additional QTLs.** Click here for file
