# Supplementary material for: Diabetes-specific genetic effects on obesity traits in American Indian populations: the Strong Heart Family Study
Source: BMC Med Genet. 2008 Oct 14;9:90. doi: 10.1186/1471-2350-9-90 (PMC2572048; doi:10.1186/1471-2350-9-90)
Supplement: Additional file 1 — Strong Heart Family Study additional QTLs. [file 1471-2350-9-90-S1.pdf]

Strong Heart Family Study additional QTLs:

Additional QTLs identified in our linkage analysis of obesity traits accounting for diabetes status were on chromosome 4 at 210 cM for weight (LOD=3.3), and at 208 cM for BMI (LOD = 4.0, between markers D4S415 and D4S1535), previously described by Almasy et al. in this population [1]. This QTL was present in both diabetic and non-diabetic individuals in stratified analysis. In addition, we identified QTLs for BMI located on chromosome 2 at 61 cM (LOD=2.1, near marker D2S367), chromosome 5 at 54 cM (LOD=1.9, near marker D5S426) and chromosome 10 at 180 cM (LOD=2.1, near marker D10S1651). The 5p13.2 and 10q26.3 QTLs were also previously described by Almasy et al.[1]. We also identified a QTL on chromosome 22 at 55 cM for WHR (LOD=2.0, marker D22S274). However, the diabetes-stratified LOD scores for BMI and WHR at these chromosomal regions were of low magnitude and not suitable for a QTL-specific analysis.

1. Almasy L, Goring HH, Diego V, Cole S, Laston S, Dyke B, Howard BV, Lee ET, Best LG, Devereux R *et al*: **A novel obesity locus on chromosome 4q: the Strong Heart Family Study**. *Obesity (Silver Spring)* 2007, **15**(7):1741-1748.
